# Work for the Wounded of the Allied Armies

**Published:** 1914-11-28

**Authors:** 


### HOSPITALS AND THE WAR.

(From our Special Correspondents.)

### Work for the Wounded of the Allied Armies.

### CAMBRIDGE: 1,250 BEDS NEARLY COMPLETED.

On Wednesday evening, last week, a Midland Railway hospital train brought to Cambridge about 173 sick and wounded soldiers from Scottish and other regiments. Not a few were men of the 6th Division, who were delighted to find themselves in Cambridge again, being, no doubt, conscious of the pleasant time which they had spent in Cambridge only a little while since. There were also a few men of the R.A.M.C. Owing to the very bad weather the trenches, in which many had been compelled to remain for hours, were in general waist-deep in water. From the 173 patients there were only about twenty-five "cot" or "stretcher" cases, the majority of the men having contracted rheumatism. A large number of the wounded received treatment in France and were now approaching a state of convalescence.

The Cambs. No. 12 Voluntary Aid Detachment, consisting of six nurses under the direction of Commandant Mrs. Ellis Long, were in readiness at the station. Upon the arrival of the hospital train, the Cambs. No. 7 Voluntary Aid Detachment (men) undertook the removal of the wounded to the various waiting rooms where refreshments awaited them, and from thence motor-ambulances and motor-cars drove them to the 1st Eastern General Hospital. The No. 7 Cambs. Voluntary Aid Detachment members were under the command of Dr. Alex. Wood and Quartermaster J. E. Scott, and they were assisted by the members of Cambs. No. 11 Voluntary Aid Detachment, Shelford, under Commandant W. L. Levereux. There were also present at the station the County Director, the Rev. C. F. Townley; Mr. J. C. Lawson, Borough Director; also Major Ballance, Surgeon R.A.M.C., Lieut. R. Porter and Sergeant Coe of the 1st Eastern General Hospital, R.A.M.C. Mr. J. S. FitzJohn, district superintendent, conducted all the railway arrangements.

The 1st Eastern General Hospital is now nearly complete and is to accommodate 1,250 wounded and sick, and at the present time about 900 are receiving attention in this hospital.

### CHATHAM DISTRICT: THE COST OF TEMPORARY HOSPITALS.

About 130 wounded have been received at Fort Pitt Military Hospital during the past week, most of whom have been defending the road to Calais.

There are to-day 2,500 wounded soldiers in the Kent Voluntary Aid Detachment Hospitals, and so well have the duties of the detachments been performed that a commendatory letter has been received from the War Office eulogising the services rendered to the wounded. The Government contribution of 2s. per head per day is not sufficient to defray the incidental and severe expenses which are necessary owing to adaptations and equipment required in institutions used as temporary hospitals; and, after a careful review of the situation, the executive committee have come to the conclusion that an additional sum of £25,000 will be required for the current year's work. Therefore, the Marchioness of Camden, the President of the Kent Voluntary Aid Detachments, is appealing for a sum of £25,000 for the current year's work. Up to the present just over £6,050 has been collected.

The detachments in the following districts have been affiliated to headquarters up to date:—

| | |
|---|---|
| Seal | Ashford |
| Beckenham | Bromley |
| Ramsgate | Chislehurst |
| Sidcup | West Wickham |
| Herne Bay | Southborough |
| Maidstone | Abbey Wood |
| Birchington | Tunbridge Wells |
| Tenterden | Farnborough |
| Biddenden | Walmer |
| Charing | Crayford |
| Sevenoaks | Broadstairs |
| Edenbridge | Cranbrook |
| Speldhurst | Westerham |
| Bexley | Sandgate |
| Hythe | Pembury |
| Teynham | Margate |
| Rolvenden | Dartford |
| Deal | Orpington |
| Tonbridge | Hawkhurst |

### LEEDS: A SOLDIER'S VIEW OF CIVILIAN HOSPITALS.

The promised accommodation of 115 beds for wounded soldiers by the General Infirmary at Leeds, since increased to 135, is being made full use of. A fortnight ago a message was received from the Military Hospital in Beckett's Park, Leeds, that the infirmary beds would be required, and within an hour forty arrived, and an intimation that at midnight—four hours later—a contingent of sixty wounded Belgians would be in. The provision for the wounded without reducing the accommodation for civilian work was arranged for by transforming the out-patient department into wards. Out-patients having received attention as usual up to within a few minutes of receiving intimation of the arrival of the wounded, necessitated very energetic work to prepare the rooms in time for the reception of the soldiers.

As the soldiers arrived they were received by the general manager, Mr. Fred J. Bray; the resident surgical officer, Mr. Flint, and his staff; and the Matron, Miss Innes. The patients were able to be put straight to bed and appeared to be very thankful to be at rest in a comfortable bed in a bright room, with the gentle attendance of the nurses and a supply of beef-tea and other nourishment. Ten days later a number were able to be sent to their respective homes on sick furlough, and fifty additional soldiers were subsequently admitted.

Many of the men who had had experience of military hospitals were strong in their expression of appreciation of the advantages and freedom of a civilian hospital. The wards at the Leeds Infirmary are not under the authority of the military hospital officers, but the wounded are received, treated, and remain entirely under the supervision of the infirmary staff until their discharge, when they are sent to the military base hospital to be dealt with. The Belgians were at first disconcerted by the fact that the dressing-gowns supplied by friends of the institution were grey, and bore strong resemblance to the German officer's overcoat!

### LEICESTER: 5th NORTHERN GENERAL HOSPITAL.

Further arrivals, including an ambulance train containing 150 wounded English soldiers, have reached the military hospital at Leicester. A number of semi-convalescent patients were thereupon transferred to the hospitals at Burton and Loughborough to facilitate the accommodation of the more serious cases arriving. Of the above convoy, with wounded men principally from the Grenadier Guards, Seaforth Highlanders, Royal Sussex, Lancashire, Wilts, Gloucester, and Duke of Westminster's Regiments, fifty were found to be "stretcher" or "cot" cases. The men had been wounded in the fighting around Ypres, and had arrived direct from the seat of war. As has been found to be the case with other arrivals, most of the men were suffering from severe shrapnel fire. The County Director, Voluntary Aid Detachments, Mr. A. W. Faire, was in charge oi the transport and ambulance arrangements, and the service he has organised has become so efficient that the transport to the base hospital is carried out with a smoothness and expedition deserving of the commendation it has received from the military authorities. Two new motor-ambulances, purchased by the County Director from contributions placed at his disposal by local residents, were found to be of great assistance. The arrangements made by Lieut.-Colonel Harrison and his staff for the reception of the men at the base hospital reached a standard only possible in an administration experienced in the handling of large numbers of sick and wounded. A further convoy of ninety sick and wounded from Flanders has also arrived.

### SALISBURY: NEW SOLDIERS' WARD AT THE INFIRMARY.

Fifty wounded men from the Front were received recently at Salisbury Infirmary. They had been wounded on the Monday night and Tuesday, and were here on the Friday of the same week. Twenty were "stretcher" cases. At the time of writing all are doing well, and some of the less severely wounded have already been able to leave on furlough. The hospital's committee are now arranging another large ward, so as to be able to offer their beds at more frequent intervals or in larger numbers.

### ST. LEONARDS NEW GENERAL HOSPITAL.

The Church Army Medical Mission's Fresh Air Home at St. Leonards is to be used as the local general hospital for poor women and children directly the St. Leonards Hospital receives the daily-expected party of wounded to whom several of its wards are to be devoted.

### BRISTOL: NEW SOUTHMEAD INFIRMARY.

Of the numerous batches of wounded soldiers who have arrived at Bristol, many of which were "stretcher" cases, the surgical cases have gone not only, as previously stated, to the Royal Infirmary, but also to Southmead, or, to give it its full title, to the 2nd Southern General Hospital, New Southmead Infirmary, Bristol. The surgical work, in fact, has been divided between the two institutions.

### BRISTOL.

Bristol has now received another big batch of 178 wounded, of which about twenty were "stretcher" cases. Seventy-two of the men were sent to the Royal Infirmary, and no fewer than 106 to Southmead. At Chipping Sodbury the Horton Voluntary Aid Detachment has secured Horton Hall as a Red Cross hospital, and some eighteen Belgian and two British wounded have been received. The Chipping Sodbury Voluntary Aid Detachment has also mobilised and equipped a hospital of five wards, capable of accommodating from twenty to twenty-five patients.

### SHEFFIELD: WHAT THE BASE HOSPITAL HAS DONE.

The Countess Fitzwilliam, of Wentworth Woodhouse, near Sheffield, has placed three beautiful state rooms at Wentworth—furnished with bedsteads and beds—at the disposal of Colonel Connell, of the Sheffield Base Hospital, for use as an auxiliary hospital. There is a private suite of apartments for the use of the *personnel*, and a special kitchen has been installed. Dr. Barr, of Wentworth, and Dr. Weatherbe, of Rotherham, have kindly offered their services. So generous has been the response to the appeal issued by Mr. F. M. Tindall that when the last convoy of 151 wounded—including sixty "stretcher" cases—came to Sheffield, the whole number were detrained and conveyed to hospital in just under an hour. Colonel Connell, acting on the hint contained in the gift of a penny from a little girl, has started a Patients' Personal Comforts Fund. The nucleus of a penny has now grown sufficiently for the officer commanding to provide with it various things which he knows the wounded need, and also in suitable cases railway fares to Sheffield. When poor parents come to the Sheffield Base Hospital their board and lodging for a couple of days or so are also provided for out of this fund. "Helping the patient in such ways as these," remarked Colonel Connell in a chat with a local correspondent of The Hospital, "meant a brighter mental outlook on the part of the patient, and will tend towards his recovery. There have to be rules and regulations, of course, but we want the patients to feel that they are being cared for in a homely way." Since the Sheffield Base Hospital opened on August 20 there have been eight convoys of British wounded received, totalling 978, and three convoys of Belgian wounded, totalling 468, while 514 members of the Territorial Force have been treated, making a grand total of 1,960.

